# Reasons for hesitancy and acceptance of COVID-19 vaccination among the Congolese population: a scoping review

**DOI:** 10.3389/frhs.2025.1647147

**Published:** 2026-02-17

**Authors:** Genèse Lobukulu Lolimo, Rodrigue Khonde, Hervé Matondo, Junias Kabele, Yannick Musawu K, Senait Alemayehu Beshah, Daniel Malik Achala, Grace Njeri Muriithi, Elizabeth Naa Adukwei Adote, Elias Asfaw Zegeye, Chinyere Ojiugo Mbachu, John Ele-Ojo Ataguba, Fadima Inna Kamina Yaya Bocoum, Serge Mayaka Manitu

**Affiliations:** 1 Department of Health Management and Policy, Kinshasa School of Public Health, University of Kinshasa, Kinshasa, Democratic Republic of Congo; 2 Environmental Health Department, Kinshasa School of Public Health, University of Kinshasa, Kinshasa, Democratic Republic of Congo; 3 National Emergency and Humanitarian Action Program, Ministry of Public Health, Hygiene and Social Welfare Programme National, Kinshasa, Democratic Republic of Congo; 4 Health System Research Directorate, Ethiopian Public Health Institute, Addis Ababa, Ethiopia; 5 African Health Economics and Policy Association (AfhEA), Accra, Ghana; 6 Economics Department, University of Kwazulu-Natal, Durban, South Africa; 7 Health Economics and Financing Division, Africa Centers for Disease Control and Prevention, Addis Ababa, Ethiopia; 8 Department of Community Medicine, University of Nigeria, Enugu Campus, Enugu, Nigeria; 9 Department of Public Health, Ethiopian Institute of Public Health, Addis Ababa, Ethiopia; 10 Department of Community Health Sciences, Max Rady College of Medicine, Rady Faculty of Health Sciences, University of Manitoba, Winnipeg, MB, Canada; 11 Partnership for Economic Policy (PEP), Nairobi, Kenya; 12 School of Health Systems and Public Health, University of Pretoria, Pretoria, South Africa; 13 Department of Health Sciences, Health Sciences Institute, Ouagadougou, Burkina Faso

**Keywords:** acceptance, hesitancy, COVID-19 vaccine, DRC, scoping review

## Abstract

**Introduction:**

Despite over 9.6 billion COVID-19 vaccine doses administered globally, vaccination access remains highly unequal. North America and Western Europe have over 50% vaccination coverage, contrasting sharply with African nations, like the Democratic Republic of Congo (DRC), which has under 10%. This scoping review explores the key factors contributing to the low COVID-19 vaccination rate in the Congolese population.

**Methods:**

We conducted a scoping review using the Arksey and O'Malley framework, searching PubMed, ProQuest, and Scopus databases for peer-reviewed manuscripts published between 2019 and 2023. Six studies met the inclusion criteria, and focused on the factors of COVID-19 vaccine acceptance, hesitancy, and access in the DRC.

**Results:**

Although surveys indicated a high willingness on the part of the people to get vaccinated, only 2.7% of the population were fully vaccinated. The primary barrier to vaccination was safety concerns, specifically, perceptions of the vaccine as new and experimental (84.4%) and fear of side effects (83.3%). Additional hesitancy factors included mistrust in vaccine effectiveness (60.4%) and a general lack of confidence (60.0%). Facilitators of acceptance included prior family vaccination, perceived risk of infection, belief in the existence of the virus, and awareness of vaccination strategies. Sociodemographic factors such as being a healthcare professional or male also positively influenced uptake.

**Discussion:**

These findings highlight the gap between vaccine willingness and actual coverage in the DRC. Addressing safety concerns and building trust through targeted outreach, especially among key professional groups, may improve vaccine acceptance and equity.

## 1 Introduction

Over 9.6 billion doses of COVID-19 vaccine have been administered worldwide, yet access remains highly unequal (1). While countries in North America and Western Europe have achieved vaccination coverage exceeding 50%, many African nations continue to fall behind. The World Health Organization set a global target of 70% coverage, but most African countries, including the Democratic Republic of Congo (DRC), have fallen short. As of 2024, only 13.4% of the Congolese population was fully vaccinated, up from 2.76% in 2022 (1–3).

Multiple factors have contributed to this disparity. Early vaccine production and distribution favored high-income countries, limiting the access of low- and middle-income nations (4, 5). Even when the vaccine became available through initiatives like COVAX, national policies and public mistrust hindered uptake (6, 7). Other obstacles concern the rapid production of this vaccine, its efficacy, ignorance of its side effects, and fear of catching the disease after being vaccinated (8–16). In the DRC, skepticism about vaccine safety, fear of side effects, and limited public awareness have been widely reported, even among healthcare workers (17).

Although prior studies have explored vaccine hesitancy globally, few have examined the specific barriers and facilitators influencing COVID-19 vaccine uptake in the DRC. This scoping review addresses those gaps by synthesizing existing literature on vaccine acceptance, hesitancy, and access within the Congolese context. The aim is to identify key obstacles and inform strategies to improve coverage in the country.

## 2 Materials and methods

### 2.1 Research design

We conducted a scoping review to identify barriers and facilitators influencing access and uptake of the COVID-19 vaccine in the DRC. This exploratory review was carried out following the analytical framework of Arksey and O'Malley (18). Arksey and O'Malley developed a five-step methodological model to guide researchers in conducting exploratory analyses. The following five-step model is proposed: (1) identification of research questions; (2) search for relevant studies; (3) study selection; (4) charting data; and (5) gathering, summarizing, and reporting the results (18).

### 2.2 Search strategy

A systematic search was conducted across three electronic databases—PubMed, ProQuest, and Scopus—covering publications from January 2019 to October 2023. We also performed a manual search of reference lists using Google Scholar, including sources in both English and French.

To enhance transparency and reproducibility the following search terms and Boolean operators were used: (“COVID-19 vaccine” OR “COVID-19 vaccination”) AND (“Democratic Republic of Congo” OR “DRC”) AND (“vaccine hesitancy” OR “Vaccine acceptance” OR “equitable access” OR “barriers to access” OR “vaccine uptake” OR “vaccination strategies”).

The inclusion criteria were peer-reviewed articles reporting on COVID-19 vaccine acceptance, hesitancy, access, or uptake; cross-sectional or other observational studies (case controls or cohort); quantitative, qualitative, or mixed studies; and a publishing date between January 2019 and October 2023.

We excluded studies without full text access as well as articles with insufficient data for extraction, editorials, letters, opinion pieces, and publications from predatory journals.

### 2.3 Data extraction and analysis

We extracted data using a standardized Microsoft Excel sheet, capturing the following variables: author(s), year of publication, study setting, data collection period, study design, population characteristics, sample size, and key findings related to vaccine access and hesitancy.

Due to the diversity of studies, a narrative synthesis approach was used to collect, synthesize, and map the literature (19). The following categories were used to classify the studies: (1) patterns of access and use of COVID-19 vaccines in the DRC, (2) barriers to equitable and timely vaccine access and uptake, and (3) strategies to address vaccine hesitancy, particularly among vulnerable populations.

### 2.4 Study appraisal

Two authors independently assessed the quality of the studies included in the review. The Joanna Briggs Institute (JBI) Quality Assessment Tool was used to assess the quality of this study (20, 21). Qualitative assessment criteria were developed with parameters ranging from low quality (<49%) to medium (50%–79%) and high quality (80%–100%) for studies that analyze trends and barriers aimed at improving equitable and timely access to and use of COVID-19 vaccines in the DRC. While no studies were excluded based on quality, the appraisal informed our interpretation of the evidence. Higher-quality studies were given greater weight in the narrative synthesis, particularly when identifying key barriers and facilitators. Lower-quality studies were included but interpreted with caution, and their limitations were noted in the discussion (19).

## 3 Results

### 3.1 Study selection

**Figure 1:**
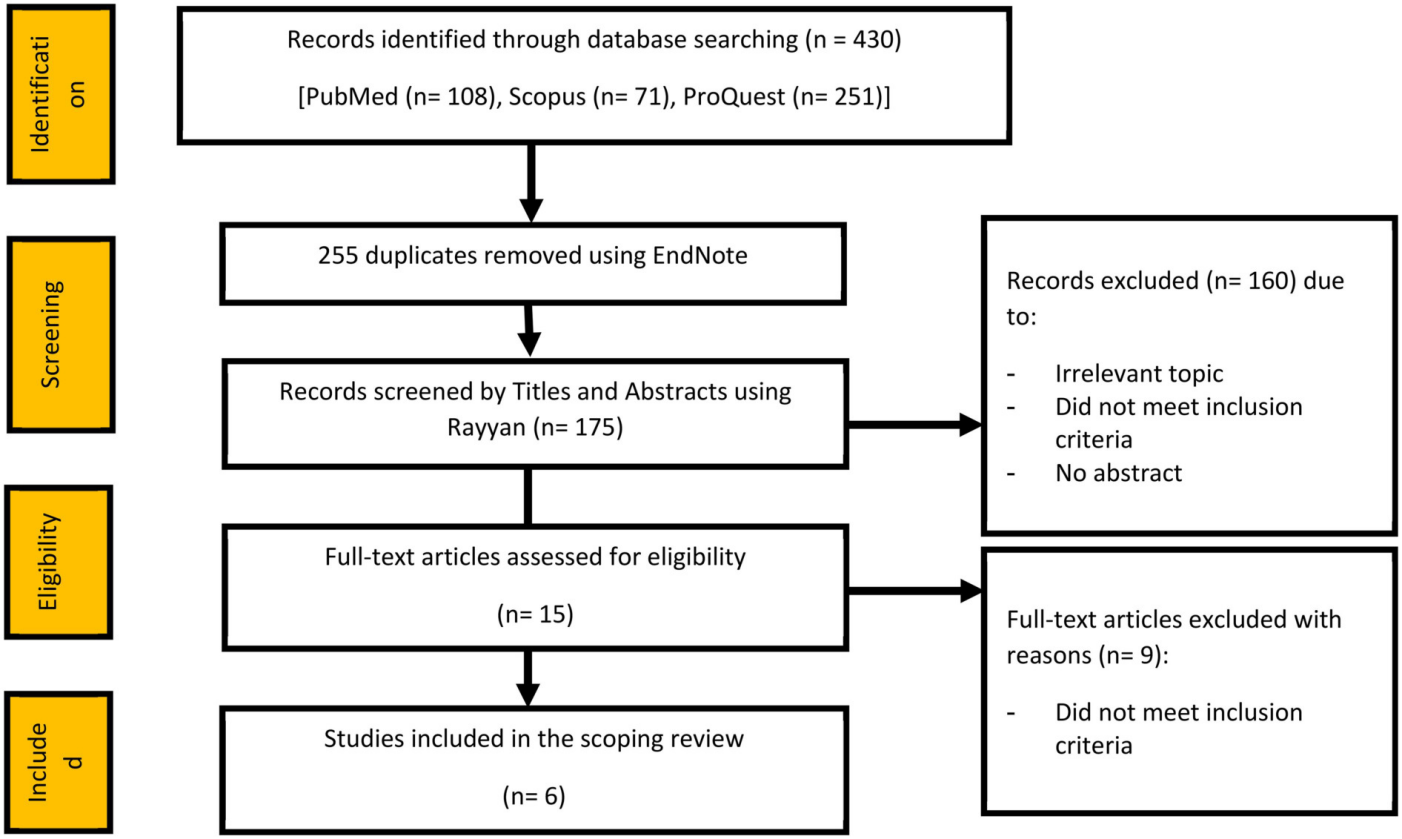
PRISMA flow diagram for inclusion process of articles in the review.

In the initial search, 430 articles were identified from searches on the databases PubMed (*n* = 108), Scopus (*n* = 71), and ProQuest (*n* = 251). We used EndNote and removed around 255 duplicate articles. After removing duplicates, 175 articles were screened by titles and abstracts using the Rayyan software. In total, 160 articles were excluded due to irrelevant topics, failure to meet the inclusion criteria, and absence of an abstract or summary of the study. Fifteen (*n* = 15) studies remained for the full-text assessment for eligibility; of them, eight full-text articles were excluded (*n* = 9) because they did not meet the inclusion criteria, resulting in six published articles for the final analysis (*n* = 6), as illustrated by the PRISMA flow diagram in Figure 1.

### 3.2 Characteristics of included studies

All six studies employed quantitative methods: Five studies used a cross-sectional design (17, 22–25) and one used a cohort design (26). Sample sizes ranged from 348 to 1,195 participants. All studies were conducted in urban or semi-urban (rural) settings within the DRC and focused on vaccine acceptance, hesitancy, or access (Table 1).

**Table 1:** Characteristics of included studies.

Reference	Title	Year of publication	Study framework (country)	Data collection period	Methodology		
					Study design	Target population	Sample size
Bateyi Mustafa et al. (25)	Determinants of parents’ intention to vaccinate their children aged 12–17 years against COVID-19 in North Kivu (Democratic Republic of Congo)	2023	DRC	1 December 2021 to 20 January 2022	Cross-sectional study	Parents of one or more children, aged 12–17 years, who lived in North Kivu	522
Whitworth et al. (24)	COVID-19 vaccine acceptability among healthcare facility workers in Sierra Leone, the Democratic Republic of Congo and Uganda	2022	DRC, Sierra Leone, and Uganda	23rd June to 27th July2021 in Goma	A multicenter cross-sectional survey	Healthcare facility workers	543 (188 in Goma, DRC)
Barrall et al. (26)	Hesitancy to receive the novel coronavirus vaccine and potential influences on vaccination among a cohort of healthcare workers in the Democratic Republic of the Congo	2022	DRC	Between 11 August 2020 and 25 August 2021	Cohort	Health workers vaccinated against COVID-19	677
Kabamba et al. (23)	Acceptability of vaccination against COVID-19 among healthcare workers in the Democratic Republic of the Congo	2020	Lubumbashi, Mbuji-Mayi, and Kamina in DRC	From 20 March through 30 April 2020	Cross-sectional study	Healthcare workers aged 18 years or older	613
Ditekemena et al. (17)	COVID-19 vaccine acceptance in the Democratic Republic of Congo: a cross-sectional survey	2021	DRC	Between 24 August 2020 and 8 September 202	Cross-sectional study	General population	413
Mashako et al. (22)	COVID-19 vaccine strategy of priority groups: perception and intention among intra-hospital health care workers in DRC	2023	DRC	From 1 5 March to 30 April 2021	Cross-sectional study	Healthcare workers	196

### 3.3 The willingness to receive COVID-19 vaccines among Congolese people

**Figure 2:**
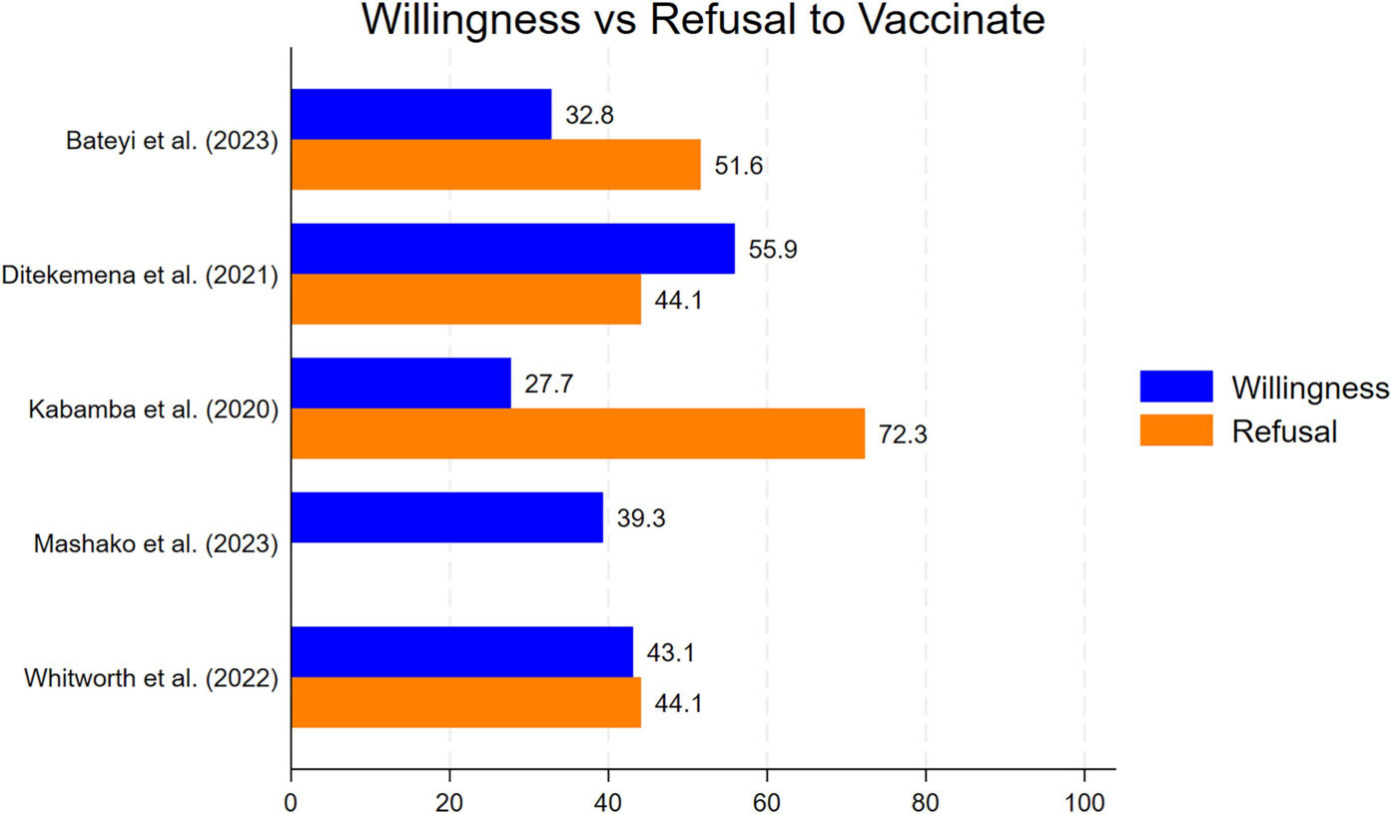
Intention to receive COVID-19 vaccine among participates in the DRC.

Five studies reported on the willingness of Congolese individuals to receive the COVID-19 vaccine (17, 22–25). This willingness varied from 27.7% to 55.9%, with the highest acceptance reported by Ditekemena et al. (55.9%). Two studies also reported refusal rates: 44.1% in Ditekemena et al. and 72.3% in Kabamba et al. (17, 23) as shown in Figure 2.

### 3.4 Barriers to COVID-19 vaccine uptake among Congolese people

**Figure 3:**
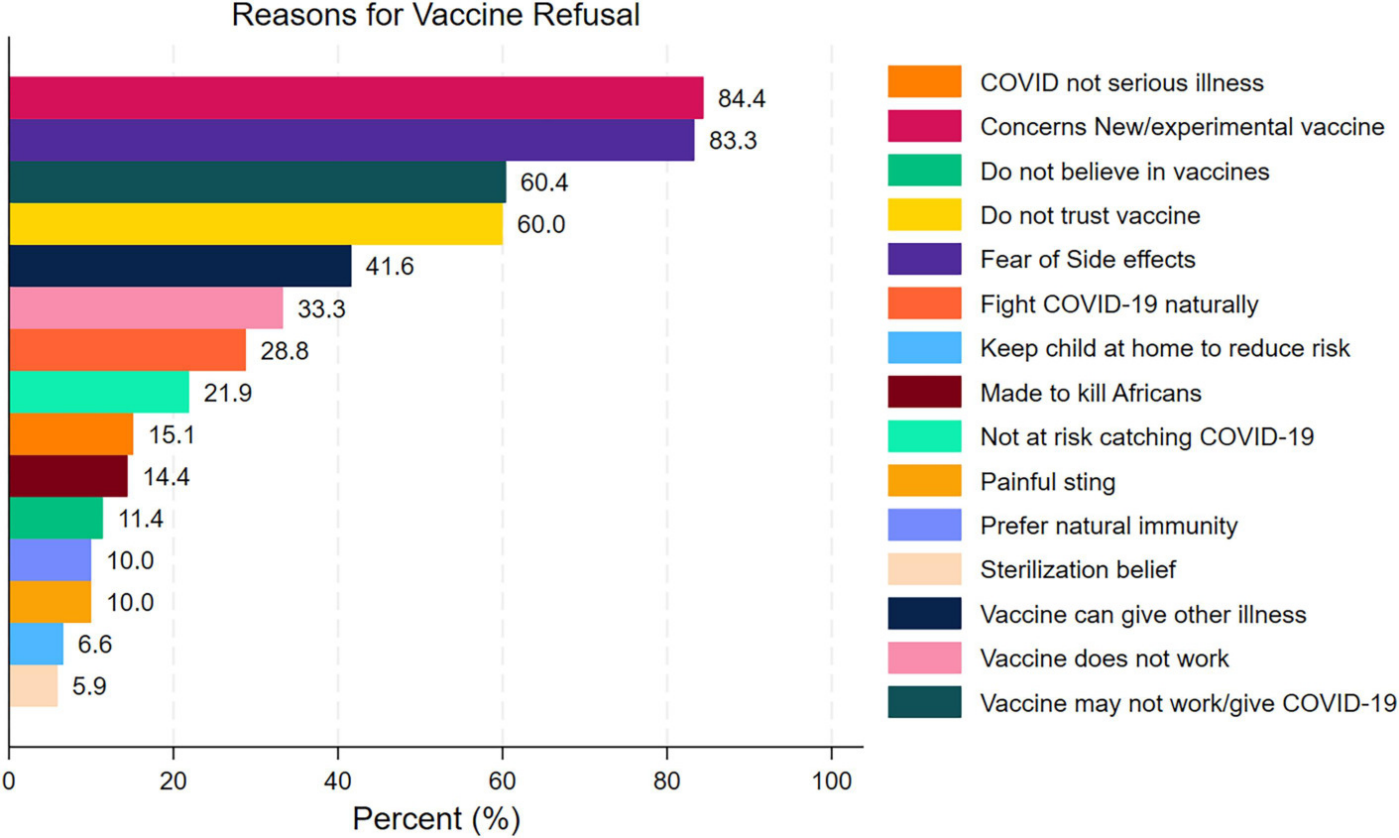
Reasons of refusal of COVID-19 vaccines among the Congolese people.

Three key barriers emerged across studies:

- Fear of side effects: This was reported by 83.3% of respondents in the study by Mashako et al. and 84.4% of respondents in the study by Ditekemena et al. (22).
- Perceived ineffectiveness: 60.4% of respondents in the Kabamba et al. study believed the vaccine would not work (23).
- Lack of trust in the vaccine: 60.0% of respondents in the Ditekemena et al. study expressed general mistrust of the vaccine (17) (Figure 3).

### 3.5 Facilitators of COVID-19 vaccine uptake

Several factors were associated with an increased likelihood of receiving the vaccination:

- Family history of vaccination: Individuals with vaccinated family members were more likely to accept the vaccine.
- Perceived susceptibility: Higher perceived risk of infection within the family increased vaccine uptake (25).
- Belief in the existence of the COVID-19 virus: Belief in the existence of the COVID-19 virus was associated with higher acceptance of the vaccine in the study by Ditekemena et al.
- Knowledge of vaccination strategy and the priority groups: Knowledge of the vaccination strategy and awareness of the priority groups were identified as facilitators by Mashako et al. (22).
- Positive attitude toward COVID-19 prevention: A positive attitude toward the prevention of the COVID-19 virus was influential in vaccine acceptance as highlighted by Kabamba et al.

**Figure 4:**
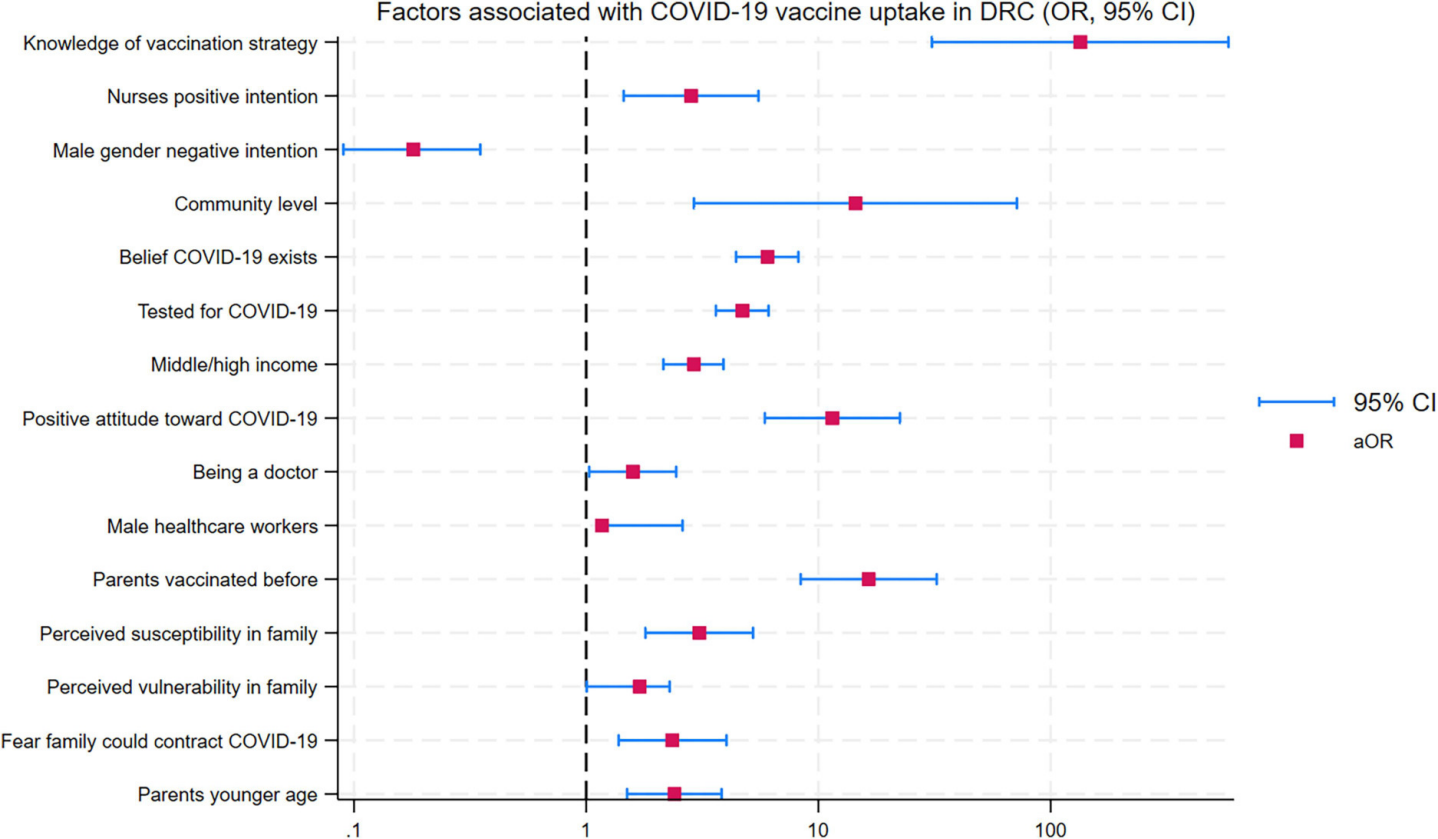
Factors associated to Uptake COVID-19 vaccines in DRC.

These findings are summarized in Figure 4.

### 3.6 Sociodemographic influences

Two studies identified sociodemographic factors influencing vaccine uptake:

- Healthcare profession: Doctors and nurses were significantly more likely to accept vaccination (22, 23).
- Gender: Male healthcare workers showed a higher acceptance rate compared to females.

These findings are summarized in Figure 4.

## 4 Discussion

This scoping review aimed to investigate the factors contributing to the low COVID-19 vaccination rate in the DRC. Six peer-reviewed studies were analyzed, primarily cross-sectional in design, with five rated as high quality and one as medium using the JBI Quality Assessment Tool.

### 4.1 The willingness to receive COVID-19 vaccine

Five studies reported on vaccine willingness among the Congolese populations, with rates ranging from 27.7% to 55.9%. Despite the expressed willingness to be vaccinated against the COVID-19 virus, only 2.7% of the Congolese population was completely vaccinated at the time of data collection (3).

These figures contrast sharply with neighboring and regional countries. For instance, Kenya reported a willingness rate of 95.1% (27), Nigeria between 50.2% and 80.9% (28, 29), and Zimbabwe reported 55.7% (30). Ethiopia, however, showed a similarly low rate of 29.2% (31).

Significantly, most DRC studies focused on healthcare workers, who tend to have higher vaccine acceptance than the general population. A study in Guinea found that 65% of healthcare workers were vaccinated compared to 31% of the general population (32). This suggests that vaccine willingness in the broader Congolese population may be even lower than reported.

### 4.2 Factors influencing COVID-19 vaccine uptake

Facilitators of vaccine uptake in DRC included family history of vaccination, perceived susceptibility to the COVID-19 virus, fear of infection, belief in the existence of COVID-19, and awareness of vaccination strategies and priority groups.

Sociodemographic factors such as being a physician, nurse, or male healthcare worker, and having higher education levels were also associated with increased acceptance (22, 26). This situation can be attributed to increased knowledge of the COVID-19 disease and its vaccine. These findings align with the results of studies in Ghana and Italy, where education, perceived risk, and social influence (e.g., seeing others receive vaccination) were key motivators (33, 34).

### 4.3 Barriers to vaccine uptake

Barriers to vaccination in the DRC were consistent across studies: (i) fear of side effects (reported by over 80% of respondents), (ii) mistrust in vaccine safety and efficacy, (iii) concerns about the vaccine being new or experimental, and (iv) limited public confidence in health authorities (17, 24, 25).

Since the end of our review period (2019–2023), several studies published in 2024–2025 have confirmed the persistence of previously identified barriers to COVID-19 vaccination uptake in the DRC, including distrust in health authorities and the vaccine, female gender, doubts about the effectiveness of the vaccine, and fear of side effects (35–40)

Similar concerns were reported in Ethiopia (hesitancy rate: 70.8%) (31) and Tanzania (63.3%) (27). Ackah et al. highlighted misinformation, media contradictions, and pharmaceutical shortages as additional barriers (41). Fernández-Sánchez et al. emphasized mistrust and limited trust among the migrant population (42).

In the DRC, vaccine hesitancy was further exacerbated by rumors, misinformation, and a lack of transparent communication during the early stages of the vaccination campaign. Toure et al. reported that in Guinea, distrust in the government was a major barrier to vaccine uptake (32).

### 4.4 Theoretical framework: understanding hesitancy

To contextualize these findings, vaccine hesitancy in the DRC can be interpreted through the lens of the Health Belief Model (HBM) and Trust Theory. The HBM states that individuals are more likely to engage in health behaviors (e.g., vaccination) when they perceive a threat, believe in the efficacy of the intervention, and feel confident in their ability to act. In the DRC, low perceived threat, doubts about vaccine efficacy, and limited trust in health systems undermine these conditions.

Trust Theory further explains how institutional trust, particularly in government and health authorities, shapes public compliance. In contexts where trust is fragile or eroded by misinformation, vaccine campaigns face significant resistance. This underscores the need for transparent communication, community engagement, and culturally sensitive messaging to rebuild trust and improve vaccine uptake.

## 5 Conclusion

This scoping review identified key factors contributing to COVID-19 vaccine hesitancy in the DRC, including lack of confidence in the vaccine development process, concerns about safety and effectiveness, and limited public awareness. Strengthening public trust and improving health literacy are essential not only for COVID-19 vaccination but also for future immunization campaigns targeting diseases such as Ebola, malaria, measles, yellow fever, and Mpox. This is particularly significant as the country faces the Mpox disease and anticipates a large-scale vaccination campaign by the Ministry of Public Health and the government.

To address these challenges, we recommend that the Ministry of Public Health, in collaboration with local health authorities, international NGOs, and community-based organizations, implement the following target interventions:

- community engagement programs to address misinformation and build trust;
- training for health workers to serve as vaccine ambassadors;
- culturally tailored communication strategies to promote vaccine benefits; and
- integration of vaccine education into schools and media platforms.

In addition, we propose conducting qualitative studies involving key stakeholders such as health workers, community leaders, and policymakers to better understand the social and structural barriers to vaccine access and acceptance.

### 5.1 Limitations

This scoping review was limited by the small number of eligible studies (*n* = 6), which may affect the generalizability of findings. Most studies involved in this review focused on health workers, potentially overlooking perspectives from the general population. Furthermore, the reliance on cross-sectional designs limits causal interpretation. These constraints highlight the need for more diverse and longitudinal research to inform equitable vaccine strategies in the DRC.

## Data Availability

The original contributions presented in the study are included in the article/Supplementary Material; further inquiries can be directed to the corresponding author.
